# Spatio-temporal analysis of tuberculosis and its correlation with the Living Conditions Index in an elderly population in Brazil

**DOI:** 10.1590/1414-431X2021e11544

**Published:** 2022-01-25

**Authors:** C.R. Mesquita, B.O. Santos, N.L.S. Soares, M.J. Enk, K.V.B. Lima, R.J.P. Souza e Guimarães

**Affiliations:** 1 Programa de Pós-Graduação em Epidemiologia e Vigilância em Saúde, Instituto Evandro Chagas, Secretaria de Vigilância em Saúde, Ministério da Saúde, Belém, PA, Brasil; 2 Laboratório de Epidemiologia e Geoprocessamento, Centro de Ciências Biológicas e da Saúde, Universidade do Estado do Pará, Belém, PA, Brasil; 3 Faculdade de Geoprocessamento, Universidade Federal do Pará, Belém, PA, Brasil; 4 Programa de Epidemiologia e Vigilância em Saúde, Instituto Evandro Chagas, Secretaria de Vigilância em Saúde, Ministério da Saúde, Belém, PA, Brasil; 5 Programa de Pós-Graduação em Biologia Parasitária na Amazônia, Instituto Evandro Chagas, Universidade do Estado do Pará, Belém, PA, Brasil; 6 Programa de Epidemiologia e Vigilância em Saúde, Instituto Evandro Chagas, Secretaria de Vigilância em Saúde, Ministério da Saúde, Ananindeua, PA, Brasil

**Keywords:** Tuberculosis, Spatial analysis, Public health

## Abstract

The aim of this study was to analyze the spatio-temporal distribution of tuberculosis (TB) in the elderly population in the city of Belém, PA from 2011 to 2015 according to the Living Conditions Index (LCI). This was an epidemiological, descriptive, ecological, and retrospective study involving 1,134 cases. Data were collected through the Information System of Notifiable Diseases (SINAN). For data analysis, we used the incidence coefficient, global and local empirical Bayesian model, Kernel density, and Kernel ratio. The construction of the LCI was based on the United Nations Development Program (UNDP) method. The incidence of TB remained the same over the five years studied. No neighborhood was found to have a high incidence of TB and a high LCI, but most of the cases occurred in the south of the city where the neighborhoods with the most precarious conditions are located. Moreover, the lowest incidence was in neighborhoods that historically had better infrastructure. Spatial analysis tools facilitate studies on the dynamics of disease transmission such as TB. In this study, it was shown that TB is heterogeneously distributed throughout the municipality. Living conditions, especially in slums, influenced TB incidence.

## Introduction

Tuberculosis (TB) is an infectious disease caused by *Mycobacterium tuberculosis*, which is found worldwide (1,2). It is considered a neglected disease and is endemic in several developing countries (2,3).

In the 2019 Global Tuberculosis Report, WHO estimated that 10 million people developed tuberculosis and 1.5 million people died from the disease in 2018, and that 3 million people believed to have tuberculosis were undiagnosed or did not receive treatment (3,4).

In the same year, 73,864 new TB cases were diagnosed in Brazil, which corresponded to an incidence coefficient of 35 cases/100 thousand inhabitants. In the state of Pará (PA), the incidence rate reached values above 51 cases/100 inhabitants in 2019, and the state also presented higher mortality rates compared to other capitals in the country (2).

Several factors affect the prevalence of TB and facilitate its spread. The prevalence of TB is associated with individual characteristics such as sex, schooling, area of residence (4), migration of individuals with TB (5), alcohol consumption, smoking, and related comorbidities such as acquired immune deficiency syndrome (AIDS) and diabetes. In addition, environmental and socioeconomic factors including poverty, household density, number of rooms in the house, basic sanitation, and other factors related to poor living conditions also affect the prevalence of TB (5-7).

Another important point is the recognition of vulnerable populations such as the elderly. The association of the elderly with TB is related to the increase of the elderly population as a consequence of longer life expectancy and the prevalence of chronic diseases in this population resulting in difficulties in detecting the disease, delaying the initiation of treatment, which in turn increases the spread of the disease and mortality. As the low immunity of this population may be related to their housing conditions and difficult access to healthcare, examining TB in this population is important (7).

In order to examine the relationship between living conditions and environmental factors and TB, it is necessary to adopt spatial analysis techniques. Moreover, understanding TB behavior in an area and its determinants is essential for implementing interventions aimed at reducing inequalities and improving treatment adherence (8). Thus, the identification of areas at different risk for TB may be essential to allow the public health system to adopt strategies tailored to the characteristics of each region and to prioritize areas with a higher incidence of the disease (9).

Thus, the objective of this study was to analyze the spatio-temporal distribution of TB incidence in the elderly population in the city of Belém, PA, according to the Living Conditions Index (LCI) from 2011 to 2015.

## Material and Methods

This was an ecological and retrospective study. The study site was Belém, the capital of the state of Pará, which is located in the northern region of Brazil and had an estimated population of 1,452,275 in 2017. Moreover, Belém has an area of 1,059.458 km^2^ with a total of 71 neighborhoods distributed into 8 administrative districts. The area has 87,754 elderly people, with an estimated life expectancy of 74 years. The municipality has a Human Development Index (HDI) of 0.746 (high) and ranks 22nd in the ranking of capital cities and sixth in the list of cities with highest HDIs in the northern region (10,11).

Data collection was done through the Information System of Notifiable Diseases (SINAN) provided by the Municipal Health Department of the municipality of Belém, PA (SESMA) from 2011 to 2015. The inclusion criteria were new TB patients who were residents of Belém with a complete address in the system aged at least 60 years at the date of notification. The total number was 1,134 cases.

Data from SINAN included information on the socioeconomic variables of the Demographic Census of 2010 and the population estimated by the Brazilian Institute of Geography and Statistics (IBGE). The variables of SINAN used in the study were: date of notification, municipality, date of birth, and full home address.

The georeferencing of TB cases was performed with digital street maps, neighborhoods, and lot and house numbers obtained from the Development and Administration Company of the Metropolitan Area of Belém (CODEM), Google Maps (https://www.google.com.br/maps/preview), and IBGE (https://www.ibge.gov.br/). In all images, information on hydrographic and neighborhood boundaries was added based on information from the 2010 IBGE census.

The following indicators were chosen for the construction of the LCI: education (non-literate population aged 60 years or older), income (two minimum wages or less of the person responsible for the household), sanitation (households with water supply and sewage not connected to the city’s network), domicile (households with ≥6 inhabitants), and slums (subnormal clusters). All indicators were built at the neighborhood level.

Slums are low-quality dwellings in urban areas that are potential sources of many epidemics (12). They were grouped into two categories: the neighborhoods that presented the classification of normal clusters were given a value of “0” and the subnormal clusters were given a value of “1”.

The LCI calculation was based on the UNDP/IPEA/FJP (1998) (13) method. First, the indices of each variable were calculated, i.e., indices were transformed to the same scale, varying between 0 and 1. The *index_i_* of the neighborhoods was calculated with the following formula:

$$index_{i}=\;\;\frac{\left(v_{i}-\;v_{i.min}\right)\;}{\left(v_{i.max}-\;v_{i.min}\right)}$$

where *v_i_* is the value of component *i* in the geographic region, *v_i.min_* is the minimum value of component *i* among neighborhoods, and *v_i.max_* is the maximum value of component *i* among the neighborhoods.

The arithmetic mean of the five indicators of each neighborhood was obtained, resulting in the LCI. Higher scores indicate that the neighborhood is at higher risk for the occurrence of TB in the elderly, that is, poorer living conditions. The result was divided into four classes, which were color-coded in maps: very low (green); low (yellow); medium (orange); and high (red).

Correlation analyses were performed to verify the association between the incidence coefficient, LCI, and each indicator (education, income, sanitation, household, and slum) using the Pearson’s correlation coefficient (r). The coefficient varies from -1 to 1; the signal indicates the positive or negative direction of the relationship between the studied variables. The closer the value is to 1, the greater the degree of statistical dependence between the variables, independent of the signal (14). The variables in the LCI are correlated in a linear trend, not being affected by outliers.

The incidence coefficient (CD_TB) of neighborhoods was determined based on the number of TB cases in a neighborhood and the population data of the Demographic Census of 2010, multiplied by 10,000. The result was grouped into four categories and color-coded: low, green (≤3); medium, yellow (>3 to 11); high, orange (>11 to 71); and very high, red (>71). As neighborhoods are subjected to small oscillations or voids, we decided to apply the Global Empirical Bayesian Model (GEBM) and Local Empirical Bayesian Model (LEBM) to reduce distortions, taking into account the population density (global and local) and spatial proximity matrix by contiguity, through local neighborhoods.

The Kernel density estimate (KDE) was used to analyze the behavior of point patterns, providing the point intensity of the process throughout the studied area by interpolation. The Kernel ratio (KR) generates a density surface by population density (15,16), which allows the comparison of cases on population density and TB density and the comparison of differences of the two analyses. The KDE and KR were applied using the parameters of quartic function and a radius of 1.5 km. The color codes for KDE and KR were: low (green); medium risk (yellow); high risk (orange); and very high risk (red).

The TerraView (http://www.dpi.inpe.br/terralib5/wiki/doku.php), ArcGIS (https://www.arcgis.com), R (https://www.r-project.org/) - ISwR and corrplot, and Excel (https://www.microsoft.com) softwares were used for data processing and analyses.

This study complied with the ethical requirements of the Declaration of Helsinki, the Nuremberg Code, and the norms of Resolution No. 466/12 of the National Health Council, and was approved by the Research Ethics Committee of the Instituto Evandro Chagas/SVS/MS (No. 1.942.983), on February 24, 2017.

## Results

**Figure 1:**
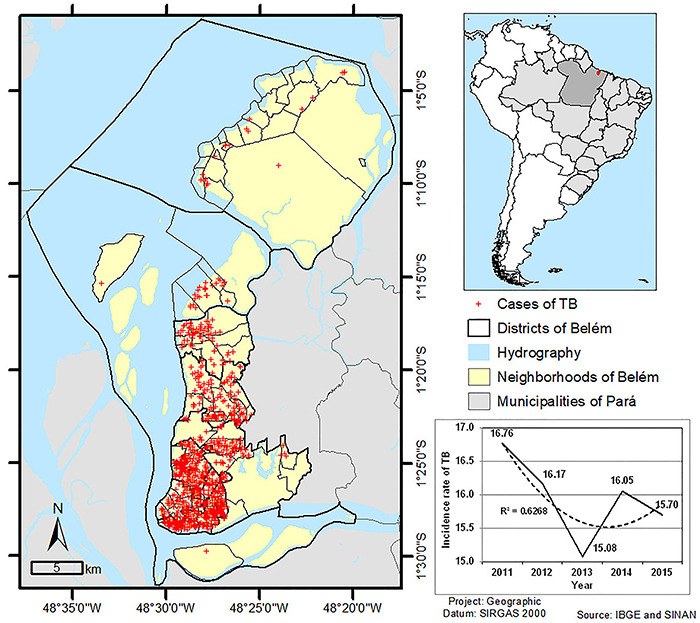
Map of Belém (larger map) with the location of the 1,134 cases of tuberculosis (TB) in the study area. Smaller map showing Brazil (light gray), State of Pará (dark gray), and Belém (red). Graph with the incidence rate of TB in the elderly population of Belém per year.

Of the cases of elderly people with notified TB, 1,134 were included in the study, and Figure 1 shows their spatial distribution.

There was a slight variation in TB incidence during the study period, with 2011 being the year with the highest incidence (16.76) and 2013 the year with the lowest incidence (15.08). The majority of the cases were aged between 60 and 69 years (56.70%), were male (60.32%), and lived in urban areas (99.12%). About 86.95% of the patients had pulmonary TB.

**Figure 2:**
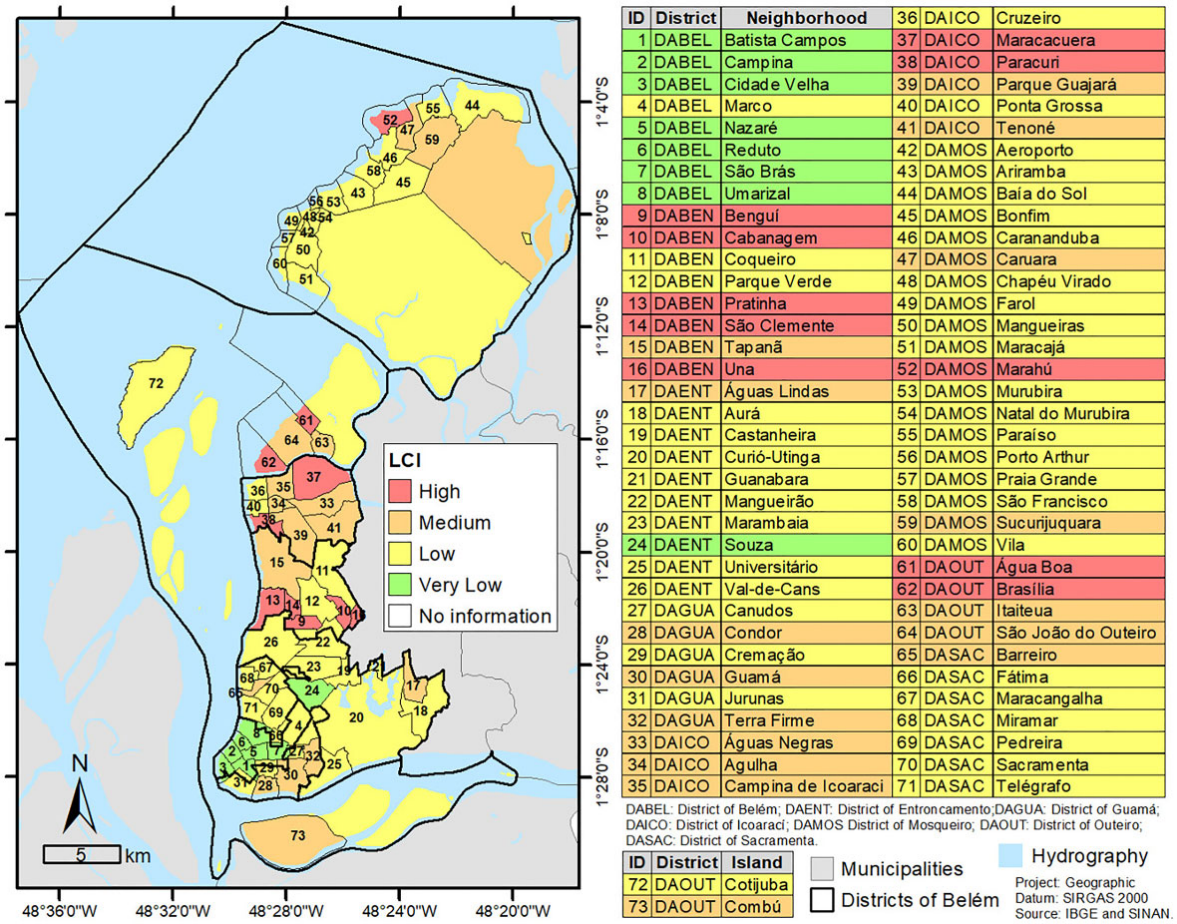
Color-coded map of Belém showing the Living Conditions Index (LCI) by neighborhoods.

Figure 2 shows the neighborhoods with the highest and lowest LCI values. The high values of LCI were noted for Água Boa and Brasília (district of Outeiro); Bengui, Cabanagem, Pratinha, São Clemente, and Una (district of Benguí); Águas Negras, Maracacuera, and Paracuri (district of Icoarací); and Marahu (district of Mosqueiro). No neighborhood with a high incidence of TB had a high LCI. The neighborhoods of São João do Outeiro and Sucurijuquara (district of Outeiro) and Barreiro (district of Sacramenta) had high incidence of TB and medium LCI. The neighborhoods with very low LCI were Batista Campos, Campina, Cidade Velha, Nazaré, Reduto, São Brás, and Umarizal (district of Belém) and Souza (district of Entroncamento).

In this study, Pearson’s r was low and with the negative signal, demonstrating that LCI and TB incidence were inversely correlated, but the result was not statistically significant (P=0.10). This may be related to the weak correlation between “education” (r=-0.08; P=0.00), “income” (r=-0.04; P=0.14), and “sanitation” (r=-0.17; P=0.00) indicators and TB incidence. In addition, these factors were inversely correlated, thus affecting the final result of the LCI. The “residency” (r=0.09; P=0.00) and “slum” (r=0.04; P=0.16) indicators presented positive coefficients, suggesting a direct relationship with TB incidence. Therefore, in this study, TB incidence was correlated with low education, poor sanitation, and a large number of people living in the same household.

**Figure 3:**
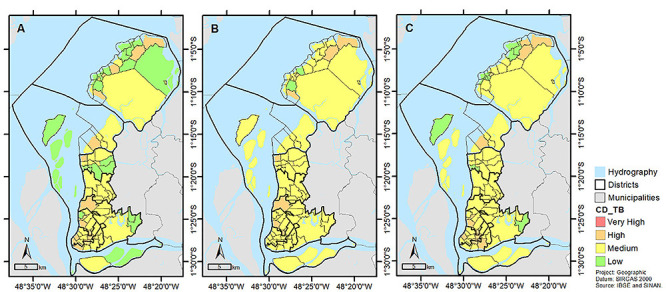
Color-coded map of Belém for incidence of tuberculosis (TB) in neighborhoods using different methods: **A**, tuberculosis incidence coefficient (CD_TB); **B**, Global Empirical Bayesian Model (GEBM); **C**, Local Empirical Bayesian Model (LEBM).

Figure 3A shows the color-coded map of the incidence coefficient by neighborhood. Neighborhoods that had high incidence coefficients were the following: Campina, Cidade Velha, and São Brás (district of Belém); Val-de-Cans (district of Entroncamento); Cremação and Jurunas (district of Guamá); Barreiro, Fátima, and Telégrafo (district of Sacramenta); Cruzeiro (district of Icoaraci); São João de Outeiro (district of Outeiro); Ariramba, Baía do Sol, Maracajá, Natal do Murubira, and Sucurijuquara (district of Mosqueiro).

After applying GEBM (Figure 3B), the following neighborhoods had a high TB incidence: Val-de-Cans (district of Entroncamento); Fátima and Telégrafo (district of Sacramenta); Cruzeiro (district of Icoaraci); Baía do Sol, Maracajá, Natal do Murubira, and Sucurijuquara (district of Mosqueiro). Moreover, the incidence in the Barreiro, Campina, Cidade Velha, Cremação, Jurunas, São Brás, and São João do Outeiro neighborhoods changed from high to medium.

Figure 3C shows the rates corrected with LEBM. The Guamá (district of Guamá) and Paraíso (district of Mosqueiro) neighborhoods appeared to have a high TB incidence for the first time. The neighborhoods of Fátima and Telégrafo (district of Sacramenta) and Baía do Sol, Natal do Murubira, and Sucurijuquara (district of Mosqueiro) remained with a high incidence. The neighborhoods of Val-de-Cans (district of Entroncamento); Cruzeiro (district of Icoaraci); and Maracajá (district of Mosqueiro) had medium incidence. The incidence changed back to a high level in the neighborhoods of Campina and São Brás (district of Belém); Cremação (district of Guamá); Barreiro (district of Sacramenta); and São João de Outeiro (district of Outeiro). A high TB concentration was observed on the border between Guamá, Cremação, and São Brás neighborhoods and on the border between Telegráfo and Barreiro neighborhoods.

**Figure 4:**
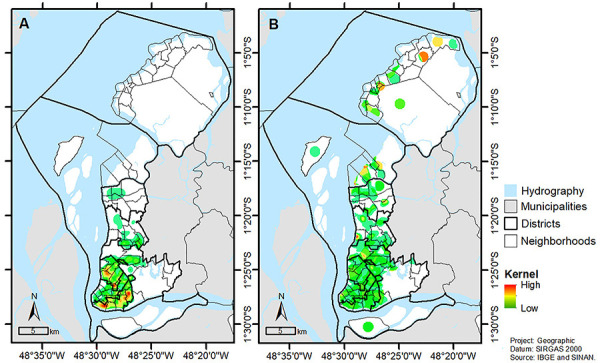
Color-coded map of Belém for tuberculosis risk using the (**A**) Kernel Density Estimate and (**B**) Kernel Ratio.

The application of the KDE (Figure 4A) showed that a cluster of very high risk formed in the neighborhoods located in the districts of Guamá (Jurunas, Condor, Guamá, and Terra Firme) and Sacramenta (Fátima, Telégrafo, Sacramenta, and Barreiro). A cluster of very high risk also formed on the border between the neighborhoods of Jurunas and Condor; Terra Firme and Guamá; Telégrafo with Sacramenta and Barreiro; and Fátima with Pedreira and Umarizal.

An interpolation of the cases was performed on the map according to distance, with an overview of the areas with the highest TB concentrations. The KDE analysis showed patterns of TB cases in a given location, allowing to highlight clusters of high TB frequency in the city.

The KR analysis takes into account the population density/incidence rate, in addition to the frequency of cases. Thus, it can be demonstrated whether the intensity of cases in a region is higher than would be expected according to the local population.

After the KR analysis (Figure 4B), the neighborhood of Pratinha (district of Benguí) and part of the Brasilia neighborhood (district of Outeiro) showed very high risk. Moreover, there were clusters of high risk in the neighborhoods of the Val-de-Cans (district of Entrocamento), São João de Outeiro (district of Outeiro), Sucurijuquara, and Natal de Murubira (district of Mosqueiro).

## Discussion

Visualization of the spatial pattern of incidence using GEBM allowed identification of a concentration of TB cases in neighborhoods in the south of Belém, near the Guajará bay, which represented an important transmission corridor (neighborhoods of Fátima, São Brás, Cremação, and Guamá). Additionally, we identified concentrations on the border between the neighborhoods of Telégrafo and Barreiro (district of Sacramenta) and in the north region (districts of Outeiro and Mosqueiro), which are areas neglected by the public policies.

Guamá is the most populous neighborhood in Belém, but it was considered a high incidence area when the LEBM was used. TB incidence in Guamá was also correlated with number of people living in the same household. The neighborhood of Guamá (district of Guamá) is influenced by the incidence in its bordering neighborhoods: Canudos, Condor, Cremação, Terra Firme (district of Guamá), São Brás (district of Belém), and Universitário (district of Entroncamento). Among these neighborhoods, only Cremação and São Brás presented a high incidence of TB cases.

The neighborhoods of Jurunas (district of Guamá) and Telégrafo (district of Sacramenta) are part of the 10 most populous neighborhoods of Belém. Cidade Velha and São Brás (district of Belém), Val-de-Cans (district of Entroncamento), Fátima (district of Sacramenta), and part of Cremação (district of Guamá) are considered by IBGE (11) as neighborhoods with the best HDI. According to IBGE, the neighborhoods that present the worst HDI are those located in the extreme south of Belém and in the districts of Outeiro and Mosqueiro (Figure 2).

Belém has considerable heterogeneity, that is, there are neighborhoods with high purchasing power and areas of poverty, which affects the spatial distribution of TB (17).

The central region has a high concentration of the oldest neighborhoods, which have the best infrastructure. The neighborhoods in the extreme south of Belém are places where unplanned growth has taken place, resulting in the creation of areas of poverty that favor the spread of communicable diseases.

The Bayesian model was used to identify areas at risk of disease transmission and to evaluate the effect of environmental and population factors. In this study, as in previous studies of spatial analysis (9,17 [Bibr B18] [Bibr B19] 20), the Bayesian model confirmed the spatial heterogeneity of TB, showing areas of higher risk together with indicators of worse social conditions. According to Lima et al. (17), this heterogeneity reinforces the need to implement targeted health interventions to the group at highest risk of contracting TB.

In recent years, Belém has experienced a surge in the incidence of TB that is almost twice that observed in the state of Pará and three times higher than the incidence in Brazil (21). In 2011, Belém was one of Brazil’s capital cities with the highest TB incidence, with a rate of 84.9 cases per 100,000 inhabitants. This may have been driven by the high population density resulting in the creation of areas of significant poverty over the years (17,22).

Belém did not meet the Millennium Development Goal for TB recommended by the WHO, which was 25.6 cases per 100 inhabitants in 2015 (23). This is of concern because there are a high number of elderly people, although the incidence of TB cases in the elderly is below the recommended level. The world population is aging, and the burden of TB continues to be important (24,25).

According to the WHO (26), the elderly population will increase fifteen times between 2006 and 2025. Elderly people are more likely to experience respiratory diseases that may have symptoms to similar TB, contributing to delayed TB diagnosis and treatment. Moreover, this could contribute to increasing TB spread and other indices (27). Therefore, TB studies of transmission patterns in different groups are required. In two studies conducted in Teresina and Piauí, the mean age of TB cases was 74 and 60 or more years, respectively, confirming the importance of studying TB in this population subgroup (25,28).

The finding that Cremação and Jurunas (district of Guamá); Fátima and Telégrafo (district of Sacramenta); and Val-de-Cans (district of Entroncamento) neighborhoods had the highest TB incidence is consistent with the findings from a study by Lima et al. (17). However, there are critical differences in some neighborhoods such as Pedreira and Sacramenta (district of Sacramenta) and Umarizal (district of Belém), areas that may have implemented TB interventions or have a smaller elderly population. Pereira and colleagues (9) reported that factors that favored TB transmission included overcrowded and poorly ventilated homes, delayed diagnosis, malnutrition, and HIV infection. The study by Silva et al. (29) also found that household density, income, and schooling may explain the differences in transmission. The greater the deficiency in a given area, the greater the TB incidence (17).

In other studies (9,17,29-31), TB was directly associated with precarious social conditions. In Belém, PA, the incidence of TB was more strongly associated with the number of people living in the same household and the slum indicator. Sanitation was not considered in this study because the municipality of Belém has low levels of basic sanitation, and therefore most of the population lives in precarious neighborhoods. Belém has 67.9% of houses with basic sanitation, ranking 1,465th among Brazilian municipalities in terms of basic sanitation (10).

In the study by Erazo et al. (30), the same indicators were used for the construction of the LCI and clusters of cases were reported in areas of social inequality and poverty. In these areas, the need for surveillance and control of the disease is even more obvious, as TB is a respiratory disease that can easily be transmitted to other regions, especially in the elderly.

As a limitation, the study had difficulties in geocoding the addresses of the cases, as it depended on the notification files, where there were often problems with the quality of completion by responsible professionals, which may lead to an underestimation of the cases. Moreover, possible migration between areas may lead to bias. For example, a person may live in one region and be infected in another.

### Conclusion

Belém is a diverse city, where neighborhoods with high purchasing power exist alongside neighborhoods with poor health and social conditions. As in other Brazilian capitals, the disordered growth in some neighborhoods, lack of planning, and population density may favor heterogeneity in neighborhoods. Belém is still considered a high-risk municipality for TB, as incidence rates exceed targets set by the WHO.

Spatial analysis tools facilitate studies on the dynamics of disease transmission such as TB. The present study showed that TB was heterogeneously distributed throughout the municipality and concentrated in areas with worse living conditions, such as the neighborhoods in the south of Belém with a high number of residents per household and slums.

The elderly population is at increased risk of TB, as older age favors transmission of the disease. Moreover, this group is often neglected, leading to delays in TB diagnosis, thereby resulting in serious complications. Therefore, new studies are necessary to analyze *M. tuberculosis* strains and their impact on disease transmission in the elderly. Interventions to prevent and control TB in this population are recommended in order to increase treatment adherence and early diagnosis.
